# Spatial Distribution of Immune Cells in Head and Neck Squamous Cell Carcinomas

**DOI:** 10.3389/fonc.2021.712788

**Published:** 2021-10-28

**Authors:** Christian Idel, Julika Ribbat-Idel, Luise Klapper, Rosemarie Krupar, Karl-Ludwig Bruchhage, Eva Dreyer, Dirk Rades, Christina Polasky, Anne Offermann, Jutta Kirfel, Sven Perner, Barbara Wollenberg

**Affiliations:** ^1^ Department of Otorhinolaryngology, University of Luebeck, Luebeck, Germany; ^2^ Institute of Pathology, University of Luebeck and University Hospital Schleswig-Holstein, Luebeck, Germany; ^3^ Pathology, Research Center Borstel, Leibniz Lung Center, Borstel, Germany; ^4^ Department of Radiation Oncology, University of Luebeck, Lübeck, Germany; ^5^ Department of Otorhinolaryngology, MRI Technical University Munich, Munich, Germany

**Keywords:** HNSCC, immune landscape, spatial distribution, TGF-β, PD-L1, PD-1, immune checkpoint

## Abstract

**Background:**

Head and neck squamous cell carcinomas (HNSCCs) have a very moderate response rate to immune checkpoint inhibitor (ICI) treatment compared to other cancer types. Lacking predictive markers for treatment response, we analyzed the immune status of HNSCC and assessed the spatial distribution of immune cells.

**Materials and Methods:**

*Via* assessing hematoxylin–eosin (H&E) stains, we divided HNSCCs by the immune cell distribution in hot, cold, and excluded tumors. For each group, each with 10 tumors, we performed serial immunohistochemical (IHC) staining of the immune cell markers, checkpoint molecules, and immune regulators.

**Results:**

The spatial distributions were different for each immune cell type, allocating regulatory T cells (Tregs) and CD11b cells predominantly in the stroma. CD4 and CD8 cells were present either in the tumor stroma or between cancer cells. Interestingly, the expressions of PD-1 (programmed cell death 1 receptor) and PD-L1 (programmed death-ligand 1) were higher in hot tumors in comparison to cold and excluded tumors. The expression of pSMAD [indicating active transforming growth factor beta (TGF-β)] was higher in excluded tumors.

**Conclusion:**

Different immune cell distribution patterns within tumors might be crucial for ICI treatment response since hot tumors have the highest expressions of PD-1 and PD-L1. TGF-β might be a key regulator for immune cell distribution and a promising therapeutic target that determines the formation of hot or excluded immune patterns.

## Introduction

Head and neck squamous cell carcinoma (HNSCC) is the sixth most common cancer worldwide (1–3). The most common therapeutic options are surgery and/or chemoradiotherapy. But these therapies are often linked to severe side effects that are hard to endure for patients. They suffer from functional impairment such as permanent voice changes or dysphagia. Surgery leads to scars and visible deformations, and a lot of patients are adversely affected by chronic pain. It has been long known that chemoradiotherapy very often leads to xerostomia, fibrosis, and necrosis of the bone and soft tissue in the head and neck region (4). Also, changes in the therapy regimens of a combined irradiation and chemotherapy only had a moderate impact on the reduction of toxicity (5, 6). Despite great research efforts, overlooking studies in the time period from 1987 up until today, the prognosis is still rather poor. In p16-negative tumors, the 5-year survival is still only 40%–60% if all tumor stages are pooled. For stages III and IV, as classified by the Union for International Cancer Control (UICC), the 2-year survival is even less since 30%–50% of patients develop local or regional recurrence, and in patients with a recurrent or a metastatic disease, the median overall survival (OS) was 10–13 months prior to the introduction of immune therapies (7–11). Tumors may originate from different locations within the group of HNSCCs, i.e., the oral cavity, oropharynx, hypopharynx, and larynx. There is growing evidence that HNSCCs of these different sites of origin differ in tumor biology. The clearest difference is seen in oropharyngeal cancers, in which human papillomavirus (HPV) has a huge impact on the OS of patients. But HPV has so far not had any impact on OS in cancers of the oral cavity, the hypopharynx, and the larynx (12). Also, HNSCCs of the different sites of origin differ in the response toward irradiation. Primary tumors of the hypopharynx have the worst response toward radiotherapy (13).

The introduction of immune therapies for solid cancers by the use of the so-called immune checkpoint inhibitors (ICIs) increased the OS rates of many patients regardless of the cancer type. The most severe impact was observed in malignant melanoma therapy, extending to cancer types such as lung cancer, where ICI treatment is very promising as well (14–16). Therefore, high hopes were set for the treatment of patients with HNSCC. The results of several phase III clinical trials showed a significant improvement compared to the standard chemotherapeutic regimen, but with mostly only a moderate improvement of the OS at the primary analysis (17–19). The 2-year follow-up data again confirm the superiority of ICI to various chemotherapy protocols, especially in patients with a higher programmed death-ligand 1 (PD-L1) expression score, but miss to achieve a stable plateau in the survival curve (20).

The results of the clinical trials have already altered the therapeutic algorithms (9), but the OS rates remain lower than those in other epithelial cancers, even other squamous cell cancers (21).

The reason for this very different impact of ICIs in the treatments of various cancers is not understood so far. Research in this field is vastly expanding at the moment.

In the clinical setting, tremendous efforts are undertaken to enroll patients in clinical trials that combine two checkpoint targeting drugs, but especially from melanoma patients, we learned that this is associated with an increased risk of severe adverse events (22, 23). The second major step comprises the use of an ICI backbone and additional targeting of a second cancer-relevant pathway.

Clinical development is severely hampered by the lack of biomarkers. Most studies are being performed as all-comer studies, lacking the right assay to predefine the most suitable patients for the drugs tested. Currently, in clinical practice, tumor response is correlated with the lymphocytic infiltrate in the tumor and the expression of PD-L1.

The immune status of HNSCC might serve as an explanation for the low impact of ICI treatment in HNSCC. Saloura et al. analyzed the genomes of two HNSCC cohorts for cytokine expression and defined two patterns, namely, high and low CD8^+^ T-cell-inflamed phenotype (24). Kulasinghe et al. gave a first impression of the distribution of immune cells within HNSCC using multiplex immunohistochemistry (IHC) to predict the response to ICI treatment. Due to the low number of samples, they have not identified a predictive marker so far (25).

Other authors divided tumors into different immune profiles, such as hot, cold, and excluded tumors, based on the infiltration of CD8^+^ T cells (26, 27). To better understand the immune profile of HNSCC, we first analyzed the immune cell distribution in tumors of primary HNSCC patients who underwent surgery as a first-line treatment in whole tissue slides. But instead of a CD8 IHC, we used hematoxylin–eosin (H&E) staining to describe the following immune status of HNSCC phenotypes:

-cold (almost no immune cells visible),
-excluded (immune cells within the tumor, but only in the stroma), and
-hot (immune cells in the stroma and between cancer cells).

In each group of 10 HNSCC patients of the hot, excluded, or cold status, we examined serial immunohistological stains. This way, we were able to establish a pattern of various immune cells linked to the degree of lymphocytic infiltrates in HNSCC. There are several markers to find first signs of regulators forming the different types of immune status.

## Material and Methods

The study was conducted in accordance with the Declaration of Helsinki, and the protocol was approved by the Ethics Committee of the University of Luebeck (project code AZ 16-277).

### Patient Selection

We established an HNSCC cohort as previously described by obtaining archived tissue samples (28). The cohort contained hot, cold, and excluded tumor tissues. We randomly selected 10 patients from each group to perform the comparative analyses, as described below. All tumors were from therapy-naive patients (PT), and none of them received ICI treatment later since ICI treatment is not yet part of the standard treatment for primary HNSCC. More details on the tumor location, tumor node metastases (TNM) stage, and later therapy are shown in **Tables 1**, **2**.

**Table 1:** Clinicopathological data of patients.

Clinicopathological parameters	*n*	Immune distribution		
		Hot	Excluded	Cold
Gender				
Male	24	9	6	9
Female	6	1	4	1
Age (years)				
≤63	18	6	7	5
>63	12	4	3	5
Karnofsky scale				
≤70	6	1	1	4
>70	16	7	7	2
n/a	8	2	2	4
Smoking				
Yes	26	7	10	9
No	3	3	0	0
n/a	1	–/–	–/–	1
Alcohol abuse				
Yes	14	4	6	4
No	15	6	4	5
n/a	1	–/–	–/–	1
Tumor site				
Larynx	6	1	2	3
Oral cavity	10	3	2	5
Hypopharynx	3	1	2	0
Oropharynx	11	5	4	2
T stage				
T1–T2	19	6	8	5
T3–T4	11	4	2	5
Lymph node metastasis				
N (0)	8	2	3	3
N (+)	22	8	7	7
Distant metastasis				
M (0)	24	9	7	8
M (+)	6	1	3	2
Pathological grade				
G1	0	0	0	0
G2	19	7	6	6
G3	11	3	4	4
UICC stage				
I–II	9	3	3	3
III–IV	21	7	7	7
p16				
Positive	9	4	3	2
Negative	21	6	7	8
Recurrence				
Yes	4	0	4	0
No	26	10	6	10

n/a, not applicable; UICC, Union for International Cancer Control.

**Table 2:** Details of all patients.

T stage	N stage	M stage	Grade	UICC stage	p16	Pack years	Alcohol	Recurrence	Follow-up (months)	Death	OP	Chemotherapy	Radiotherapy
T3	N2	M0	2	IV	Negative	30	Yes	No	56	No	Yes	No	Yes
T2	N2	M0	2	II	Positive	20	No	No	48	No	Yes	Yes	Yes
T2	N0	M0	2	II	Negative	0	Yes	No	47	No	Yes	No	Yes
T2	N1	M0	3	III	Positive	0	No	No	47	No	Yes	No	Yes
T3	N2	M0	2	IV	Negative	40	Yes	No	11	Yes	Yes	No	Yes
T4	N3	M1	2	IV	Negative	45	No	No	44	No	Yes	Yes	Yes
T1	N3	M0	2	IV	Negative	35	Yes	No	6	Yes	Yes	Yes	Yes
T2	N1	M0	3	III	Positive	0	No	No	69	No	Yes	No	Yes
T2	N2	M0	3	II	Positive	20	No	No	62	No	Yes	Yes	Yes
T3	N0	M0	2	III	Negative	30	No	No	56	No	Yes	No	No
T2	N1	M1	3	II	Positive	40	No	No	84	No	Yes	No	Yes
T1	N2	M1	3	IV	Negative	40	Yes	Yes	29	No	Yes	Yes	Yes
T2	N0	M0	3	II	Negative	10	Yes	No	84	No	Yes	No	No
T2	N2	M1	3	IV	Negative	20	Yes	Yes	15	Yes	Yes	No	No
T4	N0	M0	2	IV	Negative	30	Yes	No	83	No	Yes	Yes	Yes
T1	N2	M0	2	IV	Negative	30	Yes	Yes	10	Yes	Yes	No	Yes
T3	N1	M0	2	III	Negative	50	No	Yes	83	No	Yes	Yes	Yes
T1	N2	M0	2	IV	Negative	50	Yes	No	57	No	Yes	No	Yes
T2	N1	M0	2	III	Positive	30	No	No	64	No	Yes	No	Yes
T2	N0	M0	2	II	Positive	140	No	No	57	No	Yes	No	No
T1	N0	M0	2	I	Negative	75	Yes	No	53	No	Yes	No	No
T1	N2	M0	2	IV	Positive	3	No	No	45	No	Yes	No	No
T3	N2	M0	2	IV	Negative	40	No	No	18	Yes	Yes	No	No
T4	N2	M1	2	IV	Negative	60	No	No	6	Yes	Yes	Yes	Yes
T4	N2	M0	2	IV	Negative	60	Yes	No	0	Yes	Yes	No	No
T2	N2	M0	2	IV	Negative	17	No	No	38	No	Yes	No	Yes
T1	N0	M0	3	I	Negative	30	Yes	No	39	No	Yes	No	No
T2	N0	M0	3	II	Negative	n/a	n/a	No	0	Yes	Yes	No	No
T4	N1	M0	3	IV	Positive	60	No	No	55	No	Yes	Yes	Yes
T4	N2	M1	3	IV	Negative	35	Yes	No	7	Yes	Yes	Yes	Yes

n/a, not applicable; UICC, Union for International Cancer Control.

### Immunohistochemistry

Immune profiles (hot, cold, or excluded) were assigned after H&E evaluation by a board-certified pathologist. For 10 cases from each group, we performed IHC on 4-μm-thick sections of a formalin-fixed paraffin-embedded (FFPE) specimen after deparaffinization. We employed the IView DAB Detection Kit on a Ventana BenchMark (Roche, Basel, Switzerland). Immunostaining was performed followed by microwave-based antigen retrieval as previously described (29).

The following antibodies were used:

-CD4 [rabbit monoclonal antibody, clone SP35, ready to use (RTU); Ventana Medical Systems Roche, Oro Valley, AZ, USA]
-CD8 (rabbit monoclonal antibody, clone SP57, RTU; Ventana Medical Systems Roche)
-CD11b (rabbit monoclonal antibody, clone ER1345y C-terminal ab52478, 1:200; Abcam, Cambridge, UK)
-FOXP3 (mouse monoclonal antibody, clone 236A/E7, 1:100; Invitrogen Thermo Fisher Scientific, Rockford, IL, USA)
-PD-1 (mouse monoclonal antibody, clone NAT105, RTU; Cell Marque Sigma-Aldrich, Rocklin, CA, USA)
-PD-L1 (rabbit monoclonal antibody, clone E1L3N, RTU; Cell Signaling, Danvers, MA, USA)

### Evaluation and Scoring of Slides

For CD4, CD8, and CD11b, the percentage of immune cells and the location of positive cells (stromal *versus* diffuse) were determined. The share of FOXP3-positive cells among the CD4-positive cells was estimated. For pSMAD3, the percentage of positive tumor cells and the staining intensity were assessed and the immunoreactive score of Remmele and Stegner (IRS) was calculated. For PD-L1 evaluation, all three established scoring systems were employed, namely, the tumor positivity score (TPS), immune cell (IC) score, and the combined positivity score (CPS). For the TPS, all PD-L1-positive cancer cells were counted and put into relation to all viable cancer cells. Values are presented as percentages. For the IC score, PD-L1-positive immune cells were estimated by tumor area. For the CPS, all PD-L1-positive cells (cancer cells and immune cells) were counted and put into relation to the number of all viable cancer cells. This number was then multiplied by 100. This score has no unit. Programmed cell death 1 protein (PD-1) receptor was assessed using CPS in analogy to the CPS of PD-L1. CPS is so far the only marker for PD-L1 expression that is used for clinical decisions in HNSCC (9).

### Statistical Analyses and Graphical Visualization

Statistical analysis was performed with an unpaired *t*-test for all data presented here. *P*-values <0.05 were considered to be statistically significant. This research has made use of the statistical analyses and visualization in R software (version 4.0.2; R Foundation, Vienna, Austria; http://www.R-project.org).

We used the following software to create artwork and to edit the photomicrographs: Inkspace (version 0.92.4; The Inkscape Project c/o Software Freedom Conservancy, Brooklyn, NY, USA; https://inkscape.org/) and GIMP (version 2.10.14; The GIMP Project c/o GNOME Foundation, Orinda, CA, USA; https://www.gimp.org).

### Ethics

The study was conducted in accordance with the Declaration of Helsinki, and the protocol was approved by the Ethics Committee of the University of Luebeck (project code AZ 16-277).

## Results

### Patient Criteria

As expected for HNSCC, the majority of patients were males and middle-aged. A majority were smokers and p16-negative. They mostly presented with clinically advanced stages and lymph node metastases, and most suffered from a recurrence. Primary tumors were located in the oral cavity, oropharynx, larynx, and hypopharynx. Details are presented in **Tables 1**, **2**.

### Different Immune Cell Influx But the Same Immune Cell Proportions in the Three Immune Phenotypes

**Figure 1:**
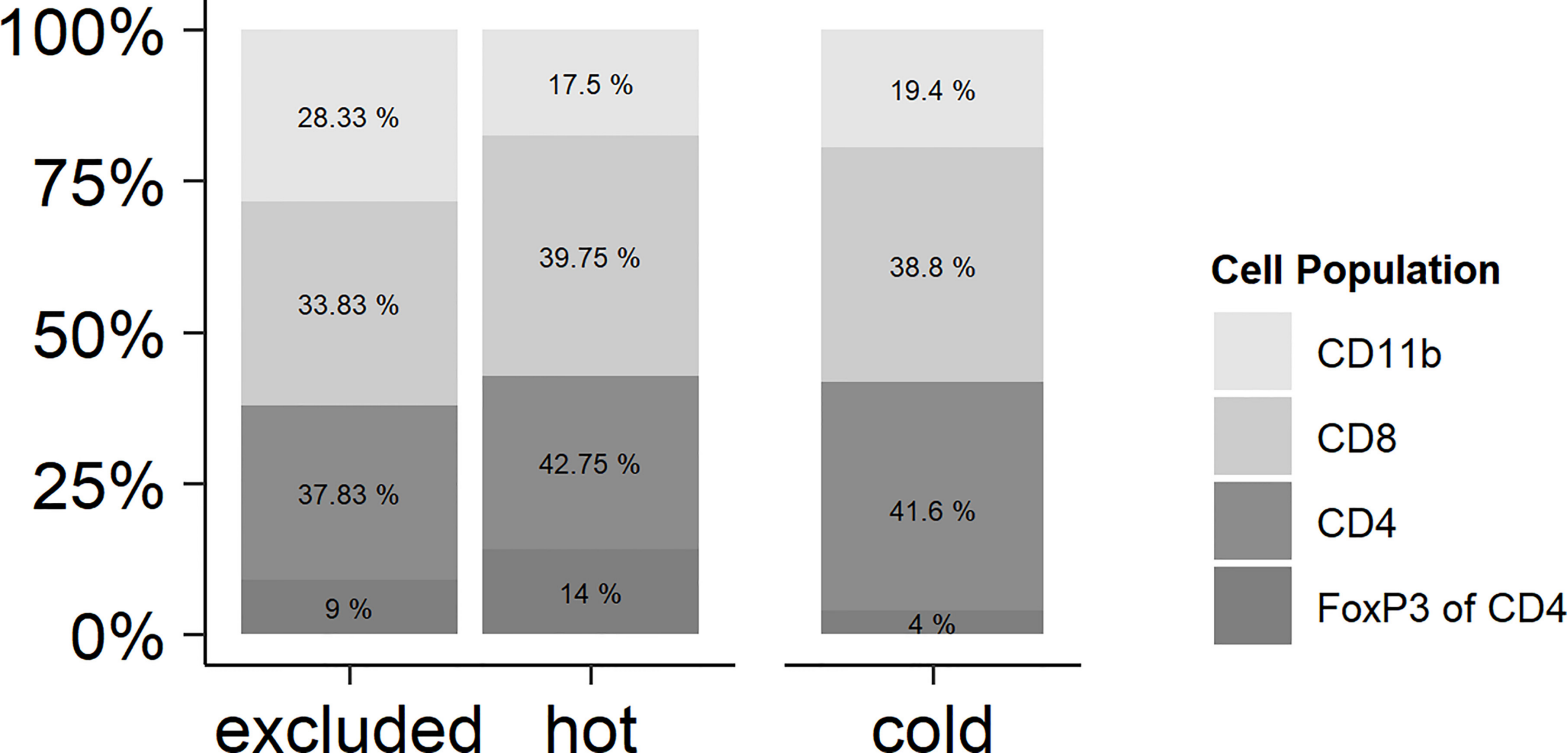
Relationships of the immune cell types in hot, cold, and excluded tumors. While the total number of immune cells differed between hot and excluded tumors on the one hand and especially cold tumors on the other hand, the relationships of CD11b-positive myeloid cells, CD8-positive T lymphocytes, and CD4 T positive lymphocytes were very similar in all three tumor immune types. There was a trend of a higher proportion of CD11b-positive myeloid cells in excluded tumors, but this difference was not statistically significant (*p* > 0.05). In CD4-positive T lymphocytes, the percentage of FoxP3-positive regulatory T cells (Tregs) was related to all CD4-positive cells. There was no significant difference in the proportion of Tregs between hot, cold, and excluded tumors.

By reading the H&E slides, we established three distinct categories of immune cell infiltrates in HNSCC. In “cold” tumors, there were only very few immune cells overall. The other categories contained more intratumoral immune cells than did the cold tumors, but differed in their distribution: hot tumors contained immune cells diffusely throughout the tumor bulk, whereas in excluded tumors the immune cells were restricted to the stromal areas. By estimating the expressions of CD8 (CD8 T cells), CD4 (CD4 T cells), FoxP3 (regulatory T cells, Tregs), and CD11b (myeloid-derived cells), it was found that there was no significant difference in the proportion of each in the three immune types (**Figure 1**). There was a trend of a higher proportion of CD11b-positive myeloid cells in the excluded tumors, but this difference was not statistically significant (*p* > 0.05).

### Different Distribution Patterns in the Three Immune Phenotypes

**Figure 2:**
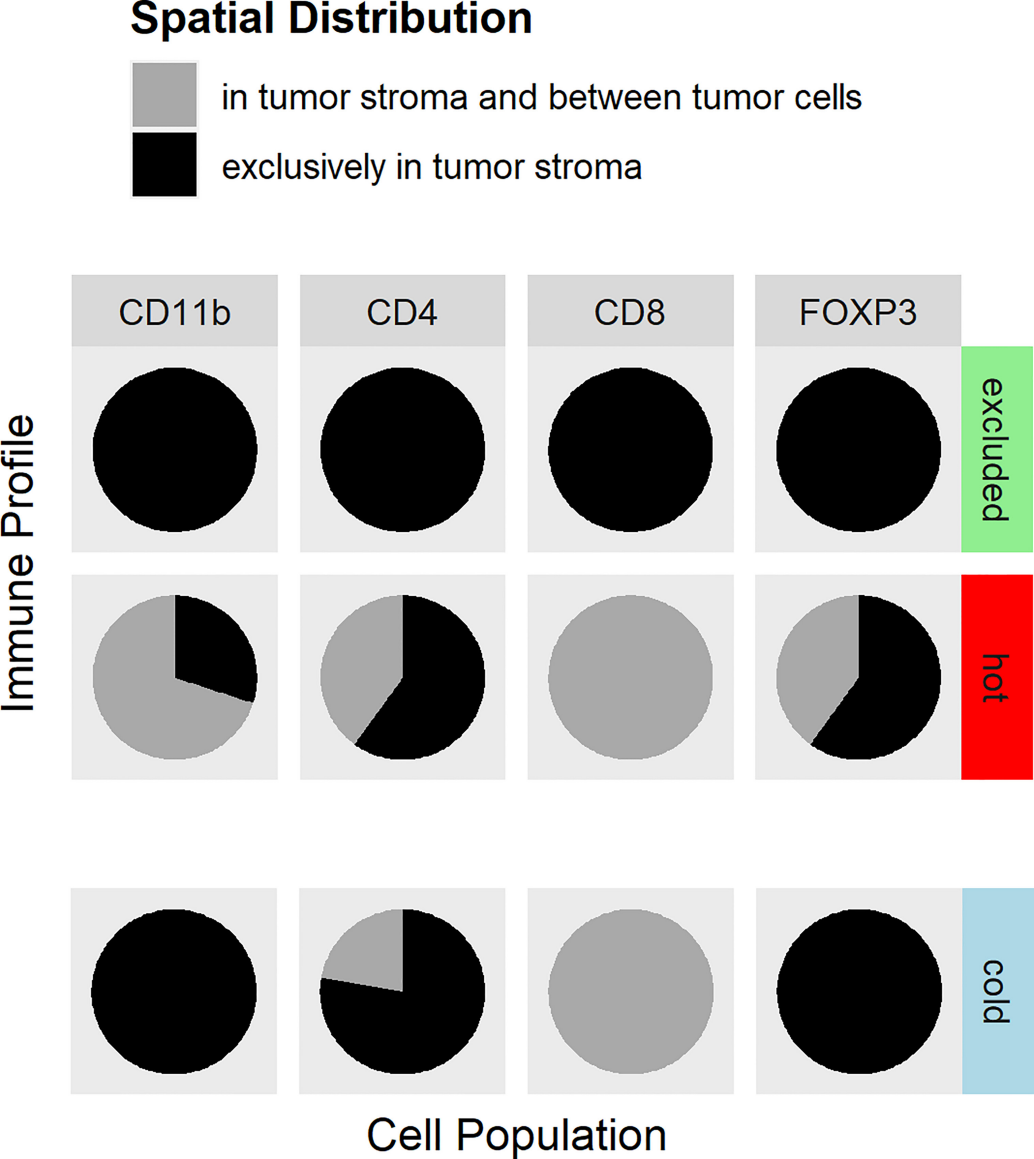
Distribution of immune cell antigens in hot, cold, and excluded tumors. The distributions of CD4 lymphocytes, CD8 lymphocytes, CD11b-positive myeloid cells, and regulatory T cells (Tregs) within tumor tissues differed between hot, cold, and excluded tumors. In excluded tumors, all four cell types are found in the tumor stroma (*black*), but not in between cancer cells. In cold tumors, there were only very few immune cells at all (indicated by the *slight offset*). The few CD8 T cells were found in the stroma and in between cancer cells (*gray*), CD4 T cells were, in most cases, in the stroma (*black*) and only in a few cases in between cancer cells and in the stroma (*gray*), while Tregs and myeloid cells were only in the stroma (*black*) in cold tumors. In hot tumors, CD8 T lymphocytes were found in the tumor stroma and in between cancer cells (*gray*). CD4 T lymphocytes and Tregs were located exclusively in the tumor stroma in most hot tumors (*black*), and in fewer cases, CD4 T lymphocytes were between cancer cells and in the stroma (*gray*). CD11b cells were in the stroma and in between cancer cells (*gray*) in most hot tumors, but in some hot tumors, they were only found in the stroma (*black*).

As mentioned above, the immune types were defined by the morphology of the H&E stain, whereas the distribution of the immune cell subtypes within the tumor was analyzed by IHC of CD8 and CD4 T cells, FoxP3, and CD11b (**Figure 1**). In cold tumors, there were only very few detectable cells of each analyzed immune cell subtype. In one tumor, there were no immune cells at all. In the other nine tumors, CD11b cells were only detectable in the tumor stroma, with CD4 T cells in the majority of cases in the stroma as well (seven tumors only in the stroma and two tumors in the tumor cells and the stroma). FoxP3 cells represented only a small fraction of the CD4 T cells, and if detectable, they were located in the stroma. In the nine cases with few immune cells, CD8 T cells were found in the stroma and in between the cancer cells. In excluded tumors, all four immune cell types were mainly in the stroma of the tumors and not in between the cancer cells. In hot tumors, the CD4 T cells and FoxP3 cells were found in 4 out of 10 tumors in the stroma and in between the cancer cells; in 6 out of 10 cases, only within the stroma. CD11b cells were detectable in the stroma and in between the cancer cells in 7 out of 10 tumors and only within the stroma in 3 out of 10 cases. CD8 T cells were located in the stroma and in between the cancer cells in 10 out of 10 tumors (**Figure 2**).

### Higher pSMAD Expression in Excluded Than in Hot HNSCC

**Figure 3:**
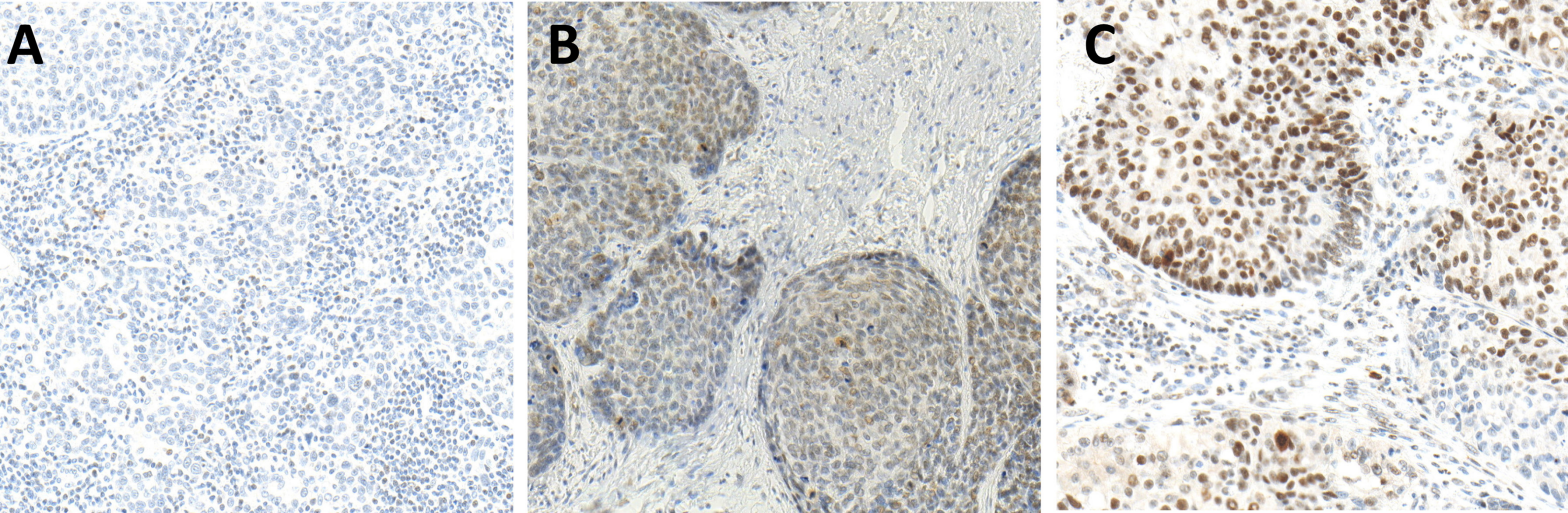
Immunohistochemistry (IHC) of pSMAD expression in hot **(A)**, cold **(B)**, and excluded **(C)** head and neck squamous cell carcinomas (HNSCCs). Hot HNSCCs **(A)** showed very low pSMAD expression, while excluded HNSCCs **(C)** had very high pSMAD expression. In cold HNSCC **(B)**, the pSMAD expression was in between.

**Figure 4:**
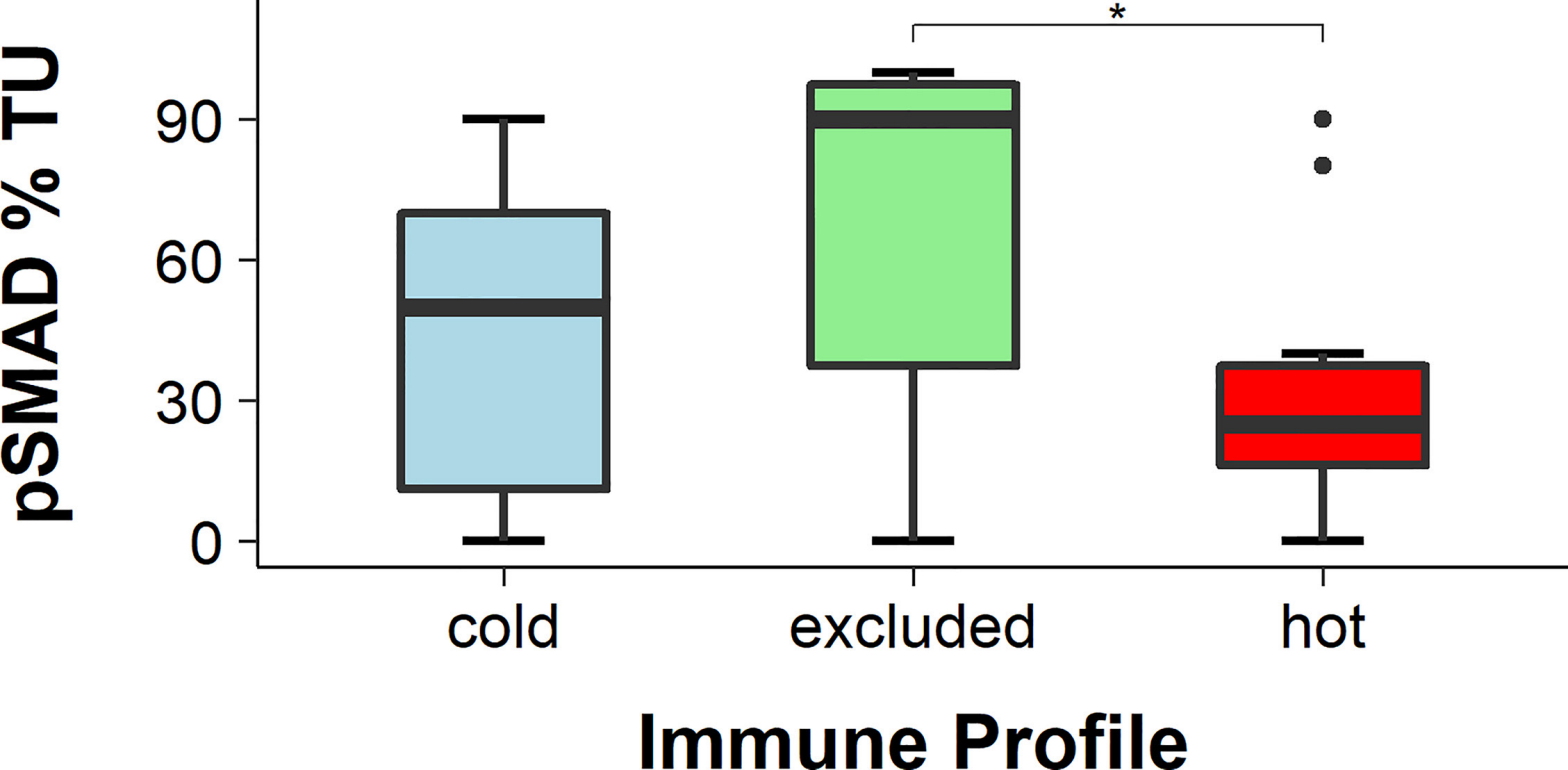
Percentage of pSMAD-positive cancer cells. The expression of pSMAD was the highest in cancer cells of excluded tumors and the lowest in hot tumors, while in cold tumors it was in between. The difference in pSMAD expression between excluded and hot tumors was statistically significant (*p* < 0.05), while the expression difference in cold tumors was not statistically significant in either of the two other tumor immune types (both *p* > 0.05), * means p<0.05.

pSMAD was measured using IHC in cancer cells and in immune cells as an indicator for an activated transforming growth factor beta (TGF-β) pathway. There was a significantly higher pSMAD expression pattern observed in the cancer cells of the excluded tumors than that in cancer cells of hot tumors (*p* = 0.0381). The expression of pSMAD in the cancer cells of cold tumors was in between that of hot and excluded tumors. Comparing the expression of pSMAD in hot and cold tumors showed no significant difference (*p* = 0.5032), and neither did the comparison between cold and excluded tumors (*p* = 0.1317) (**Figures 3**, **4**).

Furthermore, the immune cells within the sections were identified and scored based on their staining by a board-certified pathologist. The expression of pSMAD in immune cells showed no significant difference between the excluded and hot tumors (*p* = 0.2053), while there were almost no immune cells in cold tumors.

### Higher PD-1 Expression in Hot HNSCC

**Figure 5:**
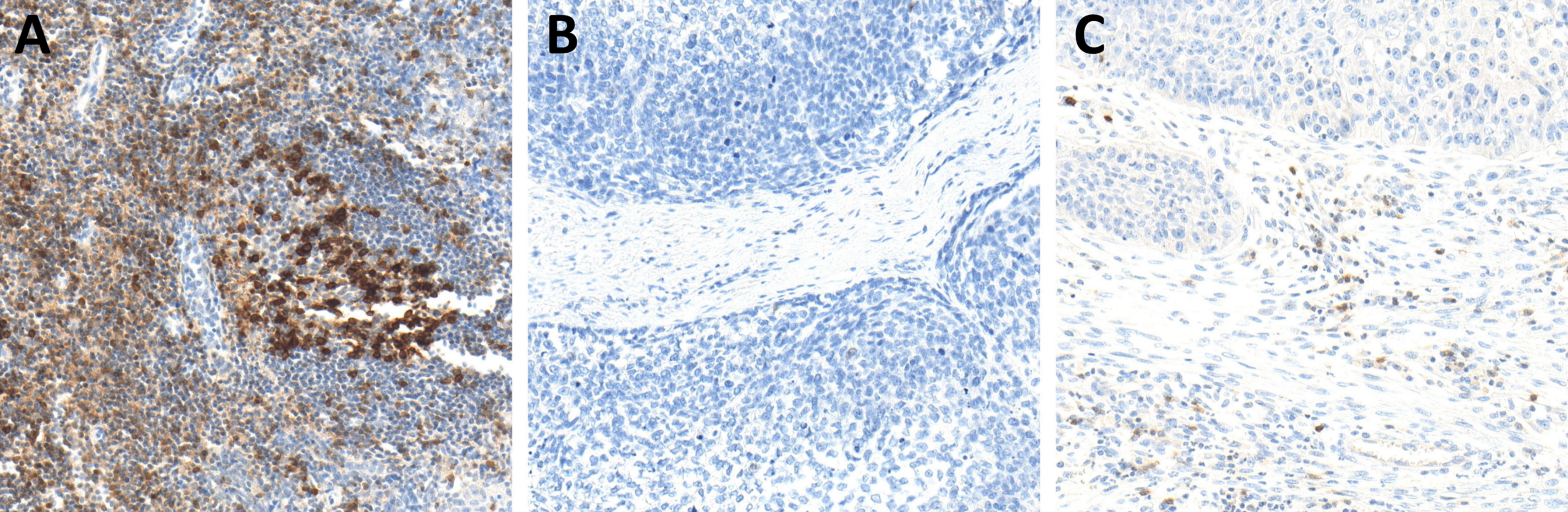
Immunohistochemistry (IHC) of PD-1 expression in hot **(A)**, cold **(B)**, and excluded **(C)** head and neck squamous cell carcinomas (HNSCCs). In hot HNSCCs **(A)**, the expression of PD-1 was very high, while it was very low in cold **(B)** and excluded **(C)** HNSCCs.

**Figure 6:**
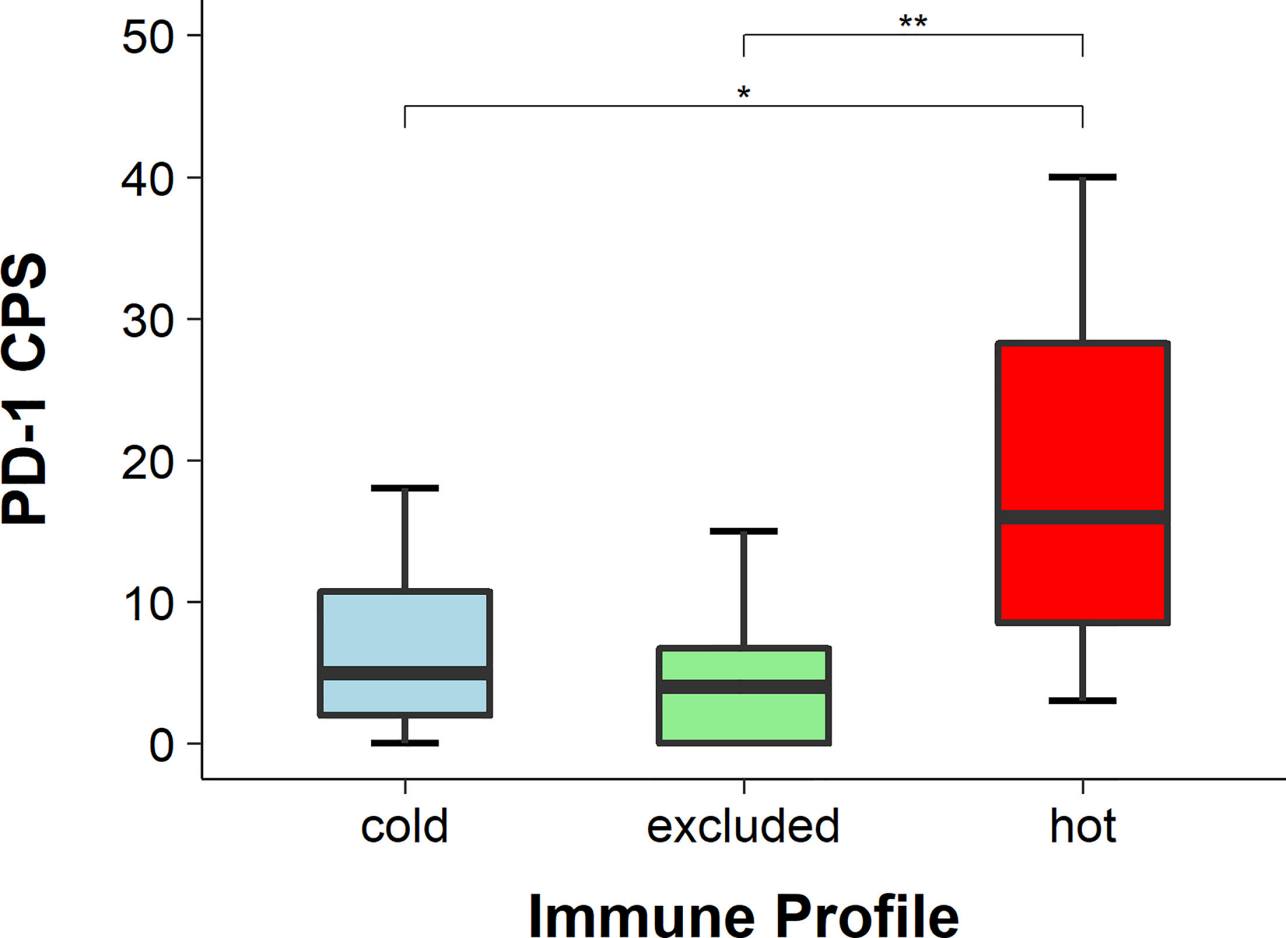
PD-1 scores in hot, cold, and excluded head and neck squamous cell carcinomas (HNSCCs; combined positivity score, CPS). The CPS of PD-1 was the highest in hot tumors and the lowest in excluded tumors, while in cold tumors it was in between. The differences in the CPS of PD-1 between hot and excluded tumors and between hot and cold tumors were statistically significant (both *p* < 0.05), while the difference between cold and excluded tumors was not statistically significant (*p* > 0.05), * means p<0.05, ** means p<0.005.

The expression of PD-1 was detected with IHC and the CPS was applied. The CPS of PD-1 was significantly higher in hot tumors in comparison with those in cold and excluded tumors (hot *vs*. excluded, *p* = 0.0027; hot *vs*. cold, *p* = 0.0304). The CPS of PD-1 in excluded tumors was not significantly different from that in cold tumors (*p* = 0.5538) (**Figures 5**, **6**).

### Higher PD-L1 Expression in Hot HNSCC

**Figure 7:**
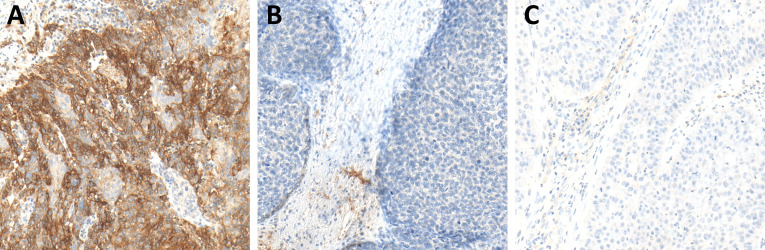
Programmed death-ligand 1 (PD-L1) expression in hot **(A)**, cold **(B)**, and excluded **(C)** head and neck squamous cell carcinomas (HNSCCs). In hot HNSCCs **(A)**, the expression of PD-L1 was very high, while it was very low in cold **(B)** and excluded **(C)** HNSCCs.

**Figure 8:**
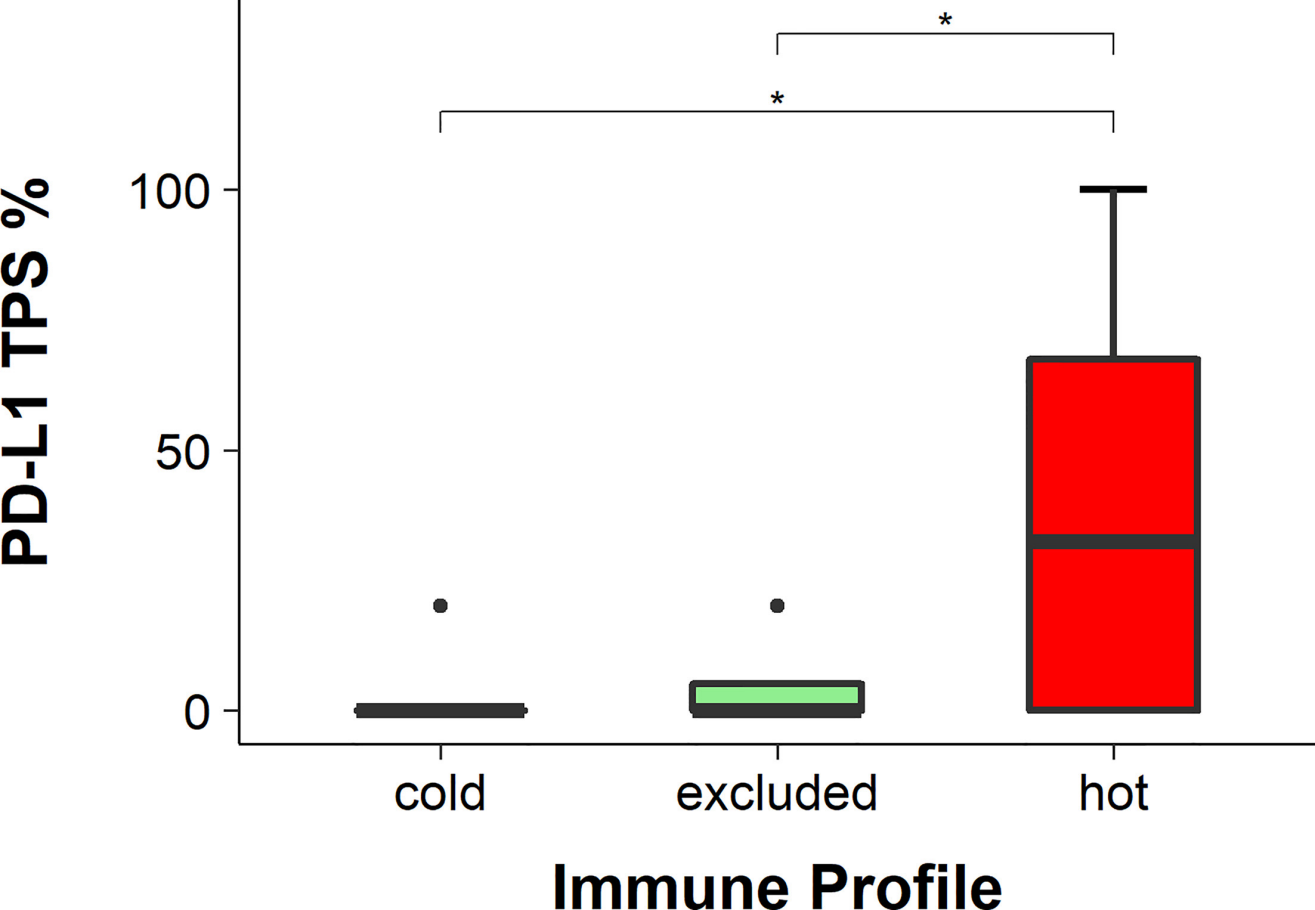
Programmed death-ligand 1 (PD-L1) scores in hot, cold, and excluded head and neck squamous cell carcinomas (HNSCC; tumor positivity score, TPS). The TPS of PD-1 was the highest in hot tumors and the lowest in cold tumors, while in excluded tumors it was in between. The differences in the TPS of PD-1 between hot and excluded tumors and between hot and cold tumors were statistically significant (both *p* < 0.05), while the difference between cold and excluded tumors was not statistically significant (*p* > 0.05), * means p<0.05.

The expression of PD-L1 was detected with IHC (**Figure 7**) and evaluated using the TPS, IC score, and CPS. The TPS of PD-L1 was significantly higher in hot tumors in comparison to those in cold and excluded tumors (hot *vs*. excluded, *p* = 0.0422; hot *vs*. cold, *p* = 0.0127). The TPS of PD-L1 in excluded tumors was not significantly different from that in cold tumors (*p* = 0.2477) (**Figure 8**). The IC score in hot tumors was not significantly higher than that in excluded tumors (hot *vs*. excluded, *p* = 0.3078; hot *vs*. cold, *p* = 0.1196). The CPS of PD-L1 was significantly higher in hot tumors in comparison with those in cold and excluded tumors (hot *vs*. excluded, *p* = 0.0011; hot *vs*. cold, *p* = 0.0085).

## Discussion

The prognosis for patients with advanced HNSCC is still very poor. Even the introduction of ICI therapy in HNSCC has not shown prognostic improvements so far (30). To better understand the differences of HNSCC in contrast to other cancer entities with a good ICI response, a lot of research was done that included RNA sequencing. Saloura et al. studied the cytokine expression patterns in HNSCC genome cohorts and proposed that the depletion of Tregs and M2 macrophages might improve the outcomes of HNSCC patients with an ICI treatment (24). It has been indicated that an IFN-γ-related profile can predict the response to treatment with the PD-1 inhibitor pembrolizumab in melanoma. This might hold true for HNSCC as well (31). In a very detailed analysis of the RNA sequencing profiles, Chen et al. described the so-called immune class of HNSCC, which contained tumors with enriched inflammatory response, enhanced cytolytic activity, and active IFN-γ signaling (32). However, RNA sequencing is still rather expensive and time-consuming. In comparison, IHC staining is more cost-effective and can be established easily without the need for expensive technical equipment. This is why, in the study presented here, we focused on IHC-based analysis to better understand the landscape of immune cells in HNSCC. In other IHC-based studies of HNSCC, the focus was on a more general description of the relation of the immune cell types rather than their spatial distribution (25, 33) or on a single location such as that of oral tongue cancers (34). The study of Meehan et al. included a mixture of PT and recurrent disease (RD) HNSCC of the tongue. As the response rate in recurrent tumors to ICI treatment is still low, some studies have tested the use of ICI in the treatment of primary HNSCC, such as KEYNOTE-689 [Study of Pembrolizumab Given Prior to Surgery and in Combination With Radiotherapy Given Post-Surgery for Advanced Head and Neck Squamous Cell Carcinoma (MK-3475-689), full text view, ClinicalTrials.gov] or ADRISK (Postoperative aRCH With Cisplatin Versus aRCH With Cisplatin and Pembrolizumab in Locally Advanced Head and Neck Squamous Cell Carcinoma, ClinicalTrials.gov). In the study presented here, we focused on treatment-naive primary HNSCC as well. The analyzed tumors were divided into three distribution patterns of immune cells. The first category, named cold tumors, had almost no immune cells in the tumor, either in the stroma or between the cancer cells. The second category, called excluded tumors, showed immune cells in the tumor, but they were limited to locations in the stroma surrounding the cancer cell areas without getting in between the cancer cells. The third type, so-called hot tumors, presented with immune cells both in the stroma and in between cancer cells.

Interestingly, mainly the CD8 T lymphocytes showed a distribution in between the cancer cells in the so-called hot tumors, while CD11b-positive myeloid cells and CD4 T lymphocytes were less frequently found in between cancer cells. By showing that in all H&E stains defining hot tumors the CD8 T lymphocytes were in the stroma and in between the cancer cells, we have provided proof that a simple H&E stain is enough for the definition of hot, cold, and excluded tumors and that CD8 IHC is not needed, as done in other studies (27). But to address the poor response to ICI in HNSCC, the expression levels of PD-1 and PD-L1 were analyzed as well. In routine diagnostics, the TPS and CPS for the expression of PD-L1 are common tools used to address the possible administration of pembrolizumab therapy in HNSCC patients (30). Also, the IC score was assessed since this score is examined for the decision about ICI treatment in lung cancer. The TPS and CPS for PD-L1 were significantly higher in hot tumors in comparison to those in excluded and cold tumors, but the IC score was not. Since the IC score only considers PD-L1 expression in immune cells—the TPS includes PD-L1 expression in cancer cells and the CPS includes PD-L1 expression in cancer and immune cells—it underlines that the main PD-L1 expression in hot tumors is in cancer cells. In a previous study, we found worse prognosis for cold tumors in comparison to those in hot and excluded tumors in a cohort of 419 HNSCC patients. This was independent of other known risk classifications such as the T stage, UICC stage, p16 expression, grading, sex, and age. Interestingly, there was no difference between p16-positive and p16-negative cancers in relation to excluded, cold, and hot cases, with 52.8% excluded, 24.8% cold, and 22.4% hot HNSCC in the p16-negative group *versus* 53.5% excluded, 23,9% cold, and 22.5% hot HNSCC in the p16-positive group (28). In the present study, we wanted to provide initial insights into the distribution of the different immune cell types in hot, cold, and excluded HNSCC.

Nivolumab and pembrolizumab are so far the two approved ICI medications for recurrent HNSCC. Since these antibodies are directed against PD-1, the expression patterns of PD-1 were analyzed as well. For this, the CPS of PD-1 was employed as PD-1 is mainly expressed on T cells, but less so in cancer cells. The binding of PD-L1 by PD-1 on T cells led to a decreased activation of the mammalian target of rapamycin (mTOR) *via* the PI3K/AKT pathway. In some cancer entities such as melanoma, PD-1 activation in cancer cells led to mTOR activation, in turn leading to cancer progression; so far, it has not been described for cancer cells in HNSCC (35, 36). Interestingly, the CPS of PD-1 was significantly higher in hot tumors in comparison to those in excluded and cold tumors. The higher PD-1 and PD-L1 expressions might make hot tumors more prone to anti-PD-1 treatment, but this needs further investigation.

Interestingly, the cancer cells of excluded tumors had a significantly higher pSMAD expression, indicating a higher TGF-β expression. TGF-β has multiple roles in physiological settings such as cell proliferation and differentiation, wound healing, and immune system, but it is very important in several pathologies, for example, in skeletal diseases, fibrosis, and cancer. In epithelial cells, it has a bifunctional role. On the one hand, it can inhibit the epidermal growth factor (EGF)-mediated cell proliferation; on the other hand, it can work synergistically with EGF in epithelial cell proliferation. Several cancer types have higher TGF-β levels than those in healthy tissues, and in several cancers, a higher TGF-β expression level is associated with cancer progression and poorer survival (37). In HNSCC, TGF-β promotes cancer cell growth. A high TGF-β expression is associated with poor prognosis and epithelial–mesenchymal transition (EMT), which might lead to metastasis (38, 39). A high TGF-β expression level also makes HNSCC cells less sensitive to cisplatin treatment by reducing the cisplatin-induced apoptosis (40). HNSCCs with high TGF-β expressions also have worse outcomes when treated with anti-PD-1 (41). In several tumor types, it was shown that TGF-β impairs the function of cytotoxic CD8 T cells (42). In the data presented here, in the immune cells of excluded and hot tumors, there was no significant difference in pSMAD expression. This might indicate that TGF-β in cancer cell areas does not directly affect the influx of immune cells into the stroma, but it is assumed that the TGF-β expression in cancer cell areas might be a barrier for immune cell infiltration in between cancer cells. In a murine model of colorectal cancer metastasis, the inhibition of TGF-β led to a higher immune cell infiltration. In this model, a single anti-PD-L1 therapy did not affect metastasis. But the combination of an anti-PD-L1 ICI with an inhibitor of TGF-β eradicated most metastasis and prolonged recurrence-free survival (43). This might also be possible in HNSCC, that the inhibition of TGF-β leads to a transformation of excluded tumors toward hot tumors. Since hot tumors have significantly higher expressions of PD-1 and PD-L1 in comparison to excluded tumors, they might be more prone to ICI treatments.

We appreciate the limitations of our study as it provides plain descriptive data and a limited number of cases. More functional analysis is needed to determine the underlying mechanisms defining hot, cold, and excluded HNSCCs and the prognostic role of TGF-β. Identifying these might lead to a better understanding of cancer progression, treatment failure, and, therefore, the optimization of therapy. With the study presented here, we wanted to give first insights into the immune cell distribution in HNSCC and a possible explanation for a high TGF-β expression being associated with worse outcomes of anti-PD-1 treatment in HNSCC. With the limited number of patients, we cannot provide any prognostic value of the TGF-β expression. But as we described worse OS for cold tumors in our previous study (28) and as all of these tumors were not treated with ICIs, we do not expect a prognostic value of TGF-β expression for the established standard therapy regimens of HNSCC. However, we are keen to learn about TGF-β analyses in future cohorts receiving ICI treatments.

**Figure 9:**
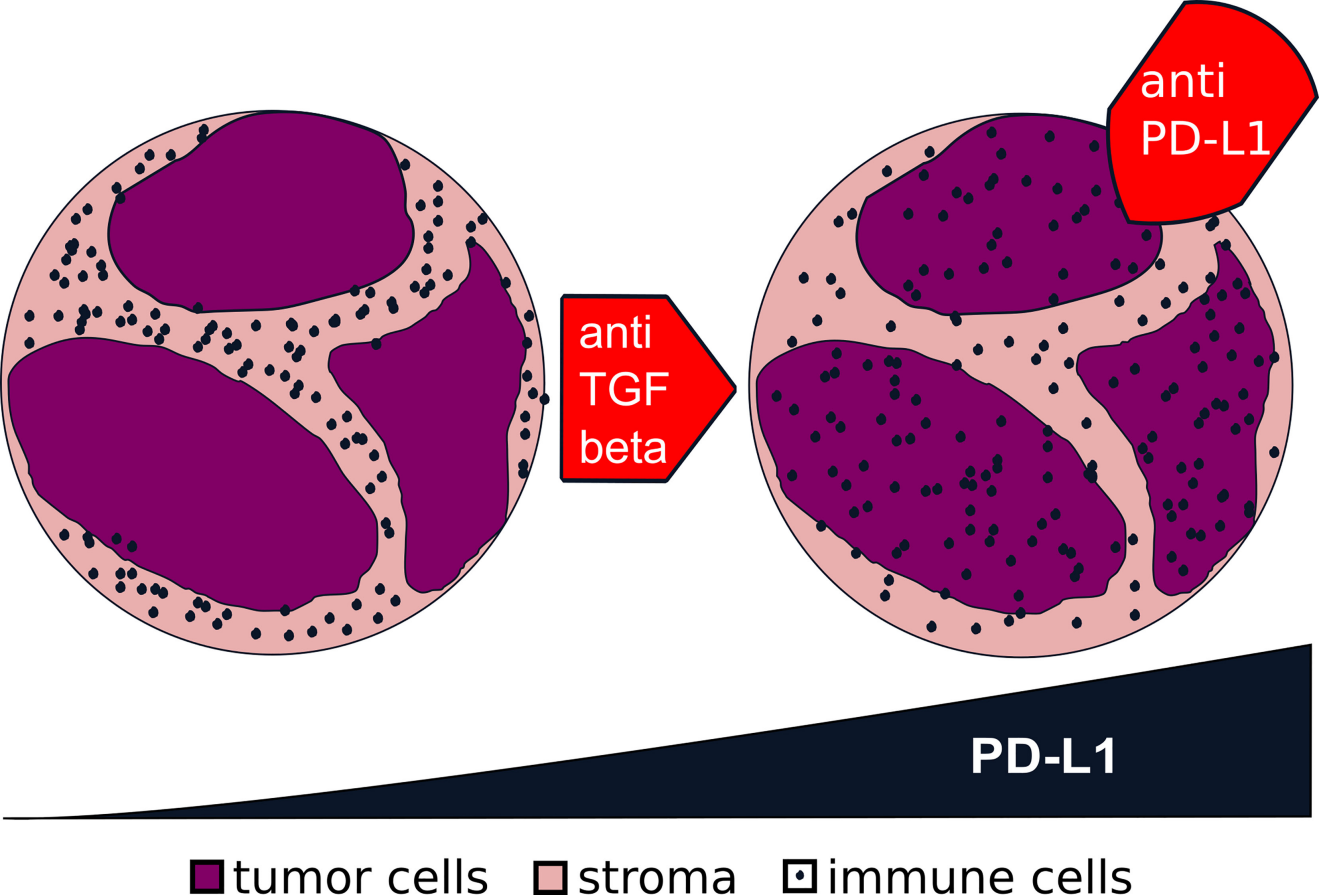
Transformation from excluded to hot tumors *via* transforming growth factor beta (TGF-β). From the data presented in this study, we generated the hypothesis for future studies: that a blockage of TGF-β in an excluded tumor (*left*) might turn it into a hot tumor (*right*). The higher expression of programmed death-ligand 1 (PD-L1) in a hot tumor might make it more prone to a blockage of the interaction of PD-1/PD-L1.

Since the introduction of ICI therapy has not had a large impact on the prognosis of HNSCCs in comparison to other solid tumor types, we still need a better understanding of the underlying mechanisms. The different distribution patterns of immune cells within tumors might be an explanation since only hot tumors do have high expressions of PD-1 and PD-L1. TGF-β might be a key regulator and will serve as a promising therapeutic target, which determines the formation of a hot or an excluded immune pattern (**Figure 9**).

## Data Availability Statement

The raw data supporting the conclusions of this article will be made available by the authors, without undue reservation.

## Ethics Statement

The studies involving human participants were reviewed and approved by the Ethics Committee of the University of Luebeck (project code AZ 16-277). Written informed consent for participation was not required for this study, in accordance with the national legislation and the institutional requirements.

## Author Contributions

SP, BW, and CI designed the study concept and approach. SP, JR-I, and CI designed the experiments. JR-I, LK, and CI performed the experiments. SP, JR-I, and ED prepared the tissues. JR-I and CI analyzed and interpreted the data, did the statistics, and wrote the manuscript. JR-I prepared the figures and created the artwork. AO provided technical advice and helped develop the methodology. JK and DR assisted with the discussion. SP, BW, RK, K-LB, and CP revised the manuscript. All authors contributed to the article and approved the submitted version.

## Funding

The study was supported by a clinical scientist fellowship of the Medical Faculty of the University of Luebeck (J26-2018) to CI and Fellowships of the Mildred Scheel Doctoral Program of the German Cancer Aid awarded to LK (grant no. 70113940).

## Conflict of Interest

The authors declare that the research was conducted in the absence of any commercial or financial relationships that could be construed as a potential conflict of interest.

## Publisher’s Note

All claims expressed in this article are solely those of the authors and do not necessarily represent those of their affiliated organizations, or those of the publisher, the editors and the reviewers. Any product that may be evaluated in this article, or claim that may be made by its manufacturer, is not guaranteed or endorsed by the publisher.
